# Stenocardia

**Published:** 1870-03

**Authors:** 


### STENOCARDIA. LECTURES OF PROF. OPPOLZER, VIENNA.

Translated by JAS. T. WHITTAKER, M.D., Cincinnati.

*Synonyms.*—Neuralgia cardiaca, angina pectoris, hyperæsthesia plexus cardiaci, brustbraune, herzklemme.

By this disease is understood an extremely violent pain extending from the cardiac region, where it originates, out under the sternum, radiating very often into the left upper extremity, and also in other directions, and attended with a feeling of greatest anxiety, "approaching death."

Heberden was the first to describe this disease accurately and to display its nervous character. What nerves of the cardiac plexus are affected, however, still excites a diversity of opinion among authors; in all probability all the nerves entering in its composition participate. In favor of this view speaks the fact that in stenocardia disturbances of nerve force occur both in the motor and sensitive spheres, as evidenced in the former by the abnormal activity of the heart, and in the latter by the violent pain. Romberg denotes stenocardia as a hyperæsthesia of the cardiac plexus. Bouillard as a neuralgia of the phrenic nerve. Heberden as a cramp of the heart, etc.

*Etiology and Pathological Anatomy.*—If we are so far in the light with the entity of stenocardia that we may regard it as a nervous disorder, for which its sudden and paroxysmal appearance and disappearance speaks, we are still in obscurity as to its first cause. So much is certain, that it attacks generally a riper age, and unequally oftener the male than the female sex, and that in by far the great majority of cases a valvular disease of the heart, namely, of the aortic valves, is concomitant. There may be consequently a certain connection between stenocardia and valvular disease. Not seldom the autopsy has revealed an ossification of the cardiac arteries, and upon this Home, Parry, Lussana, and Friedreich have laid great stress, or an atheromatous affection of the heart, generally the aorta, an aneurism of the latter, or a cicatricial formation in the heart, an adhesion with the pericardium, fatty or lardaceous degeneration of the cardiac tissue; in short, circumstances which implied a tension or other irritation of the cardiac plexus, or the ganglions imbedded in the heart's substance, and in all these manners has the origin of the stenocardic attacks found an explanation.

On the other hand, conditions have been found on section where anatomy was unable to detect the slightest abnormity; and yet during life a stenocardia was present in high degree. Gout is also considered an etioliological circumstance by many, which seems indeed in individual cases justifiable. Finally, there remains still to mention the so-called reflex stenocardia, wherein there is no disease of the heart or its adnexa, but some affection of an organ far removed therefrom. So the occurrence of this disease is occasionally remarked with affections of the uterus, the kidneys, liver, or stomach, etc., the stenocardic attacks clearly emanating from disease of one of the organs mentioned and terminating with their cure. A few such cases have occurred here in connection with catarrh of the stomach, and a proper treatment for this malady has been equally efficacious in removing the accompanying stenocardia.

*Symptoms.*—Stenocardia occurs, as already mentioned, in paroxysms, with intervals of variable duration perfectly free from pain. The attacks occur either during the hours of occupation, or so to speak, spontaneously in the night during sleep, or after meals.

*The paroxysm* may be recognized by the following characteristics: sudden and extremely violent pains are felt in the substernal region, generally exactly corresponding to the cardiac region, and as already partly intimated, radiating, in the rule, into the left, but often also into the right arm and in the maxillary region, in rare cases finally extending down into both lower extremities. The pains in their quality are either burning or lancinating, and are generally attended with a high degree of oppression, a feeling as if the throax was laden with a heavy burden. Great compression exists, the patient is compelled to observe the greatest possible rest, the face is pale, seldomer cyanotic, sunken, covered with cold perspiration and bears an expression of greatest anxiety. The heart's activity during the attack in the majority of cases is extremely tumultuous and violent, so that the throax is forcibly shaken, the impulse at the apex is far more extensive, and on auscultation, nothing can be observed of the sounds or accidental murmurs present, only simply the clinking stroke of the organ on the parieties of the chest. The pulse at the same time observes no relation to this vehement activity, remaining small even scarcely palpable, which circumstance may find its explanation in the fact that in consequence of the violent action of the walls of the heart the proper filling of its cavity and the propulsion of the pulse wave is hindered. The pulse is besides very often also irregular and intermittent. Finally it occurs quite often that in spite of an increased frequency of the cardiac contractions the impulse remains remarkably feeble and limited to a small area; this is the case when the heart is in the condition of fatty degeneration, or an extensive adhesion with the pericardium is present.

The condition of respiration during a stenocardic attack is well worth mention. This in most cases is short, violent, and whistling, although a quiet deep inspiration can be executed without much effort; this dyspnœa must therefore, as Heberden has justly observed, be only regarded as a result of the feeling of great anxiety.

Only exceptionally are the patients able to observe a dorsal decubitus; in the rule they are compelled to sit or stand, very often grasping any firm objects in their vicinity. They hasten also not seldom to the window when the attack occurs in a room and seek in this manner satisfaction for the feeling of hunger for air. Toward the close of the attack evacuations occur, or even vomiting, escape of intestinal gases, discharge of feces and evacuation of urine of a water-like clarity. The duration of an attack varies from one minute to half an hour. After the attack a feeling of debility and depression remains for a time, a disturbance of the disposition, and in many cases also a dull pain in the chest or shoulder, attended in the arm with a feeling of debility or slight anæsthesia.

*Diagnosis.*—The diagnosis of stenocardia presents generally but little difficulty. The chief symptoms which characterize the disease are the substernal pains, their radiation toward the shoulder, the neck, and the arms to the elbows, or often even to the fingers; further, the extreme feeling of anxiety and the sense espiratory oppression. Stenocardia might be confounded with the asthmatic attacks following pulmonary disease; a more accurate examination, however, would soon dissipate such an error. Even so, a proper knowledge would forbid a mistaken diagnosis in the cases of the hysterical asthma of emales, or the asthma of hypochondriasis. From simple nervous palpitations, hyperkinesis cordis, stenocardia may be differentiated by the absence in the former of the substernal pains and their radiations, as well as of the feeling of "approaching death," with its attendant thoraicic oppressioos.

*Prognosis.*—The prognosis of this disease is a varied one. Should a demonstrable alteration in the heart or the vascular system (atheromatous disease) lie at its basis, the prognosis is unfavorable; and in those cases especially where the attacks occur after short intervals, and display an increase in their intensity. The danger then consists in the facts that a sudden death may occur during the paroxysm, or the patient may become marasmic and die of debility. When no organic disease exists, however, when the paroxysms are not particularly intense, and the intervals are longer, then is the prognosis not unfavorable, as the malady in such cases may exist for many years and not bring the patient into much danger. Unfortunately our experience compels us to reckon the cases of stenocardia without demonstrable lesions among the exceptions. Favorable is the prognosis finally when the disease is of reflex origin, if the affection of the organ inducing it be amenable to treatment

*Therapy.*—The therapy is divided into that of the attack and that of the interval.

*During attack.*—Above all things is the greatest possible rest to be observed, which however is generally the case, as every motion increases the difficulty. If the heart's action be very much increased, administer a dose of opium $\frac{1}{2}$ gr. or its preparations, morph. acet. $\frac{1}{6}$ $\frac{1}{4}$ $\frac{1}{3}$ gr., to which also gr. j–ij quin. sulph. may with advantage be added. Digitalis is not among the favorite remedies, since small doses are powerless in relief and larger doses, with the exhaustion of the cardiac nerves already to be feared in consequence of the enormously increased activity, might easily induce a paralysis of the organ. Besides we may attempt a counter-irritation with exciting clysters, mustard plasters, etc., tepid maniluvia and pediluvia, to which a handful of farina seminum sinapis may advantageously be added, and ice applications may be laid over the precordia to quiet the excited action. When the sense of oppression is very severe chloroform inhalations may finally be attempted, but always with the greatest caution, and never to the extent of loss of consciousness.

Should symptoms occur during a stenocardic attack which intimate great feebleness of the cardiac action, or should the paroxysm excite fainting or other evidences of debility, an excitant treatment must be introduced. Wine, acetic or sulphuric ether, alone, or with camphor (℞. æth. acet., ʒj.; camphor, gr. iij.; s. gtt. viij., in dessert-spoon of water), liquor ammon. anisat., liquor cornu cervi succinat., warm envelopments to the cold hands and feet, frictions of the forehead and temple, with eau de cologne, etc. In those cases, where appearances of death exist, reanimative efforts are to be undertaken and persevered in with patience, since conditions of apparent death, according to experience, do not belong to the rarities.

*During interval.*—When the paroxysms have displayed anything of a periodicity, quinine stands at the head of all remedies for commencing the attack; and in those cases, too, wherein the paroxysms display no typical return, quinine is still to be employed, and resort is only to be had to other remedies when this has failed; other remedies recommended are the different preparations of zinc, cuprum ammon, nitras argenti, or the tr. arsenic Fowleri; argentum, however, only to be employed when all other remedies have failed, as a *dernier resort*, on account of its occasional evil effects. A case occurred here, wherein a patient affected with psoriasis was subjected to arsenical treatment for several months, the consequence of which was that a paralysis of a l four extremities occurred, and remained defiant of all remedies until death. Symptoms of chlorosis or anæmia indicate a roborant treatment—and here the iron remedies play an important role. In general, the therapy of this disease calls always for a regard of its exciting cause, which is especially to be sought out in the so-called reflex forms.

Besides the proper medication, certain rules of caution are to be recommended. Patient must refrain from all excesses and from exciting drinks; he shall abstain from hot food, the ingesta to be nutritious and easily digestible; mental excitement strongly forbidden. He shall remain in-doors in bad, particularly windy, weather; when the circumstances admit it, he is to be sent to a climate without extremes. The grape cure exercises, not rarely, an alleviating influence, particularly when the malady is not too severe; the regular cold water cures, on the other hand, are not to be recommended, while simple cold washings, or even cold baths, are permissible and beneficial; swimming, however, is most decidedly to be forbidden.—*Cincinati Lancet and Observer.*

